# The Sense of Agency in Driving Automation

**DOI:** 10.3389/fpsyg.2019.02691

**Published:** 2019-12-03

**Authors:** Wen Wen, Yoshihiro Kuroki, Hajime Asama

**Affiliations:** Department of Precision Engineering, The University of Tokyo, Tokyo, Japan

**Keywords:** sense of agency, sense of control, driving automation, joint control, robotics

## Abstract

Driving automation has been developing rapidly during the latest decade. However, all current technologies of driving automation still require human drivers’ monitoring and intervention. This means that during driving automation, the control by human driver and by the driving automation system are blended. In this case, if the human driver loses the sense of agency over the vehicle, he/she may not be able to actively engage in driving, and may excessively rely on the driving automation system. This review focuses on the subjective feeling of agency of the human driver over the vehicle in such situations. We address the possible measures of agency in driving automation, and discuss the insights from literatures on the sense of agency in joint control, robotics, automation, and driving assistance. We suggest that maintaining the sense of agency for human driver is important for ethical and safety reasons. We further propose a number of avenues for further research, which may help to better design an optimized driving automation considering human sense of agency.

## Introduction

The sense of agency refers to the experience of controlling one’s own actions, and through this, external events (Gallagher, 2000). In past decades, the experience of agency has received great attention in the fields of cognitive science, neuroscience, philosophy, and engineering. In the fields of cognitive science and neuroscience, researchers have aimed to clarify the processes that underlie this subjective experience and to propose reliable methods to measure it. In the field of philosophy, the sense of agency is tightly linked to the core matter of self-consciousness. In the field of engineering, the rapid development of human-computer interaction technology has brought challenges in interface design. How do people perceive their agency in the context of human-machine interaction and collaborative control? The sense of agency is crucial for human behavior and mental states. For example, the sense of agency is critical for motor control and action selection (Murata et al., 2016), and people favor actions that are linked to a sense of agency over those that are not (Karsh and Eitam, 2015). More importantly, the subjective feeling of responsibility raises important legal and ethical issues when human control and machine control are blurred during automation assistance (Limerick et al., 2014; Nyholm, 2018).

In this review, we focus on the field of driving automation. The technology of driving automation has gained explosive attention in the past decade, and the technology is maturing for practical use. The Society of Automobile Engineers (SAE) International defined six levels of driving automation from level 0 to 5 as follows, depending on the extent to which the driver is involved in driving (SAE International, 2014) (cited from SAE J3016):

1. *SAE Level 0*: No automation. The full-time performance by the human driver of all aspects of the dynamic driving task^1^, even when enhanced by warning or intervention systems.
2. *SAE Level 1*: Driver assistance. The driving mode-specific execution^2^ by a driver assistance system of either steering or acceleration/deceleration using information about the driving environment, with the expectation that the human driver performs all remaining aspects of the dynamic driving task.
3. *SAE Level 2*: Partial automation. The driving mode-specific execution by one or more driver assistance systems of both steering and acceleration/deceleration using information about the driving environment, with the expectation that the human driver will perform all remaining aspects of the dynamic driving task.
4. *SAE Level 3*: Conditional automation. The driving mode-specific performance by an automated driving system of all aspects of the dynamic driving task with the expectation that the human driver will respond appropriately to a request to intervene.
5. *SAE Level 4*: High automation. The driving mode-specific performance by an automated driving system of all aspects of the dynamic driving task, even if a human driver does not respond appropriately to a request to intervene.
6. *SAE Level 5*: Full automation. The full-time performance by an automated driving system of all aspects of the dynamic driving task under all roadway and environmental conditions that can be managed by a human driver.

The commercial extent of driving automation is currently at or below Level 2, likely not only because of technical issues but also because of the legal and ethical role of “the driver” (Nyholm, 2018). In particular, many new commercial cars provide Level 1 driving assistance, in which the control of the human driver and system are blended, raising an important question: where is the threshold over which human drivers no longer feel a sense of agency and realize that the car is not under their control? In fact, the better the automation, the less drivers engage in controlling the car. For example, some Tesla drivers were reported to fall asleep when using Autopilot (Loeffler, 2019). Drivers may respond to SAE Level 2 automation as if it were SAE Level 3 automation if they do not feel a sense of agency over the vehicle. A recent study showed that even when the vehicle’s supervision successfully reminded drivers to hold the wheel and look at the road, people still failed to engage in driving and were unable to prevent the vehicle from crashing into a conflict object (Victor et al., 2018). This study showed that the key component of driver engagement is cognitive.

In this review, we aim to provide a fundamental understanding of the concept and measures of the sense of agency along with the role of the sense of agency in engineering fields, such as robotics. Furthermore, we discuss the possible link between the sense of agency and the development of driving automation, suggesting potential research focuses and the direction of an optimized driving automation system which accounts for the human sense of agency. We also reviewed literature that provided useful insights for measuring and maintaining the sense of agency in driving automation. Finally, in the discussion of the importance of the sense of agency in driving automation, we suggest two directions for future research on this topic.

## The Sense of Agency: Definition and Concept

The sense of agency refers to the subjective feeling that one is controlling their own actions, and through them, external events (Haggard and Chambon, 2012). This definition actually contains two layers (Wen, 2019). The first refers to control of one’s own body according to one’s own will and can be called body agency. Body agency is developed over the course of a lifetime. It is usually reliably held and considered a default form of control among the healthy population, but may be impaired by some mental disorders such as schizophrenia and apraxia (Moore and Fletcher, 2012). On the other hand, the second layer refers to the feeling of controlling external events and can be called external agency. The sense of agency that many studies have examined in laboratory experiments is external agency. Compared to body agency, external agency integrates more high-level processes (Synofzik et al., 2008, 2009; Moore and Fletcher, 2012) and can be less robust and more flexible (see Wen, 2019 for more discussion). In the case of driving automation, only external agency is considered relevant.

The comparator model was the most dominant account of the sense of agency in recent decades. It suggests that the sense of agency emerges from comparisons between predicted sensory feedback, which is generated through motor commands, with actual sensory feedback (Blakemore et al., 1998, 2002; Frith et al., 2000; Haggard, 2017). When the two match, people feel a sense of agency. When there is a mismatch (i.e., prediction error), the sense of agency diminishes. The comparator model is useful in explaining body agency but is usually insufficient to account for external agency. For example, Wegner et al. (2004) showed that people felt an illusionary sense of agency over another’s movement when they heard instructions describing the movements prior to the other’s motion. In Wegner et al.’s (2004) experiment, there were no efference copies of motor commands, but the sense of agency emerged. In such a case, the sense of agency was probably generated from higher level processes. Higher level processes that influence the sense of agency include regularity detection (Wen and Haggard, 2020), inference (Moore et al., 2009), goal achievement (Wen et al., 2015b,c), and social interactions (Beyer et al., 2017; Ciardo et al., 2018). Particularly important among these high-level processes is goal achievement. People feel a strong sense of agency if they successfully achieve an intended goal, even when low-level sensorimotor processes raise prediction errors (Wen et al., 2015c). In the case of joint driver-automation control, a driver’s sensory predictions usually do not perfectly match the motion of the vehicle. The critical component for maintaining a sense of agency may be a comparator at a higher level: whether the vehicle’s movement matches the driver’s intention.

## Measurement of the Sense of Agency

The measurement of a subjective feeling is always challenging for scientists. In psychology, the most common method is self-reporting. Experimenters ask questions such as “Do you feel that you controlled the object on the screen (yes or no)?” and “How much control did you feel that you have over the motion of the object (rate between 1 and 7)?” To answer such questions, participants usually need to retrospect on what they had done and felt to make a judgment. Self-reporting a sense of agency may of course introduce individual judgment bias. However, with sufficient sample size, the individual difference in judgment criteria can be diluted. The subjective judgment of agency includes (1) binary judgment (no control vs. control); (2) categorical judgment (e.g., classifying trials in different conditions into self-control, biased control, and other-control); and (3) rating (e.g., 5-, 7-, or 9- point rating, or continuously adjusting a rating bar). The different methods of self-reporting may result in slightly different response patterns. For instance, employing a rating system with many levels may result in a more linear response compared to binary judgment. Importantly, when using subjective report, researchers also need to consider human tendencies in classification behaviors. Specifically, when people notice that there are several different experimental conditions, they tend to attribute different responses to them (i.e., rating points), even if their sense of agency is comparable in all of the conditions. For example, in many studies using delayed feedback, agency ratings gradually decreased along with the increases in delay, despite the fact that participants’ perception of the causal relation between their actions and the feedback is intact (Wen, 2019).

Beyond self-reporting, sensory attenuation and intentional binding are two methods to implicitly measure the sense of agency. Sensory attenuation refers to the phenomenon that a self-produced stimulus feels less intensive than an externally produced stimulus (Blakemore et al., 1998). For example, experimenters ask participants to rate the intensity of a stimulus, such as a touch. The intensity rating of a self-produced stimulus is usually lower than that of an other-produced stimulus. Therefore, sensory attenuation can be used as an index of whether people feel a sense of agency over the stimulus. The fact that one cannot tickle oneself is a well-known phenomenon of sensory attenuation. The predicted sensory feedback of a self-touch is generated prior to the actual action (i.e., touch of one’s own skin), and when the actual sensory feedback is received *via* the skin, it undergoes a process of “central cancellation” in the brain and therefore, this stimulus tickles less than another’s touch (Blakemore et al., 1998). Self-produced touch, sound, and visual stimuli have all been reported to be less intense compared to externally produced stimuli (Synofzik et al., 2006; Gentsch and Schütz-Bosbach, 2011; Timm et al., 2014; Gentsch et al., 2015; Kilteni and Ehrsson, 2017). However, the mechanism of sensory attenuation remains controversial. Besides the theory of central cancellation, the pre-activation theory suggests that when neurons are prepared to receive the feedback and are pre-activated before the actual sensory feedback, resulting in a larger activation in the baseline but a smaller difference between the actual activation and the baseline (Roussel et al., 2013). Such smaller differences from the baseline, rather than weakened processing, explain sensory attenuations (Roussel et al., 2013).

Nevertheless, sensory attenuation is a robust phenomenon for measuring self-produced feedback and is useful as an implicit measure of the sense of agency in some circumstances. However, sensory attenuation is not suitable as a graded measure, as it shows a fading effect. Specifically, when the self-produced feedback is delayed longer, the sensory attenuation becomes weaker, but the effect of delay does not further increase along with increased action-effect intervals when the effect is delayed by more than 200 ms (Blakemore et al., 1999). In short, sensory attenuation is not suitable for measuring the sense of agency when the effect may be delayed by more than 200 ms. Furthermore, because sensory attenuation is considered to occur in low-level processes, it often does not correlate with subjective judgments of agency when high-level processes are involved (Dewey and Knoblich, 2014; Wolpe and Rowe, 2014).

The intentional binding effect is another well-known implicit measure of the sense of agency. It refers to the phenomenon in which the perceived time codes of an action and its consequence are attracted to each other when people feel a sense of agency over the consequence (Haggard et al., 2002). The experimenter asks participants to make a temporal judgment of their own actions and the subsequent stimulus, such as “When did you press the button?” and “When did you hear the tone?,” under the hypothesis that when people have a sense of agency over an external effect (e.g., a tone), the time perception of their action would shift toward the timing of the effect, and the time perception of the effect would also shift toward the timing of the action. The original procedure used to study intentional binding involves four separate stages. In each trial, participants watch a clock hand rotating with a period of 2,560 ms (this length is from Libet et al. (1982) original work, but the exact length is not essential) and report the onset time of an event. In the *voluntary action* block, participants perform an action (e.g., press a key) at a time of their own choice, and judge the time at which they performed the action by reporting the number on the clock face. Participants’ response in the voluntary action block provides a baseline of participants’ time perception of an action when the action does not cause any effect. In the *stimulus only* condition, participants are presented with a stimulus (i.e., a tone) at a random time and report the onset of the stimulus. This provides a baseline of time perception for an event when the event is not caused by participants themselves (i.e., participants feel no sense of agency over the events). In the *action-operant* condition, participants perform an action, and the action causes an event at an interval of 250 ms. Participants only report the onset of their action. On the other hand, in the *stimulus-operant* condition, participants perform an action that causes an event, and they report the onset of the event. The two operant conditions indicate participants’ time perception of their voluntary action and the event caused by the action, which is compared with the above two baselines. The extents to which the action and event are perceived to shift toward each other are called action binding and effect binding, respectively.

Intentional binding is useful in measuring the sense of agency while minimizing the influence of judgment bias, as it basically involves a perceptual task. The most important feature of the binding is that the effect disappears if the action is involuntary (Haggard et al., 2002). Haggard’s group and many other researchers worldwide have used intentional binding to measure the sense of agency in many tasks. Some researchers also compared intentional binding with other measures of sense of agency but found diverging results. Some studies indeed found that intentional binding coincides with explicit agency judgments (Braun et al., 2014; Imaizumi and Tanno, 2019), but others reported that the two measures do not involve the same self-attribution processes (Ebert and Wegner, 2010; Dewey and Knoblich, 2014; Saito et al., 2015; Wen et al., 2015a). This may due to the different processes underlying the sense of agency that intentional binding and explicit judgment measure. Explicit judgment of agency may integrate more high-level processes, including inference, expectation, and bias, while intentional binding may reflect the influence of causal relationships on time perception. A recent study proposed a Bayesian inference model to estimate the sense of agency through the influence of the accuracy of timing estimation on the intentional binding effect (Legaspi and Toyoizumi, 2019). This Bayesian approach provides a possible way to measure the sense of agency trial by trial using the intentional binding effect while controlling the noise in time perception. In addition, besides the original clock paradigm, researchers also used a simpler method to measure the time perception of the action-effect interval, by asking participants to estimate the interval rather than report the onset time of the action and effect (e.g., Ebert and Wegner, 2010; Wen et al., 2015a). The interval estimation method cannot distinguish *action* binding from *effect* binding, but can be used for continuous action tasks (Wen et al., 2017b) and reduce participants’ loads.

## Physiological Signals Linked to the Sense of Agency

All of the measures mentioned above have some obvious limitations. The explicit judgment of agency could easily be biased. Sensory attenuation is not suitable for continuous actions and events. Intentional binding is very noisy and requires many trials to compare the mean values between conditions, so it cannot be used for single-trial measurements. Most importantly, all the above methods require responses from participants and therefore involve continual interruptions of actions. However, psychologists and neurosciences have since explored the usefulness of measuring electroencephalogram (EEG) signals.

EEG signals are weak electric signals we can measure on the scalp using precise electrodes and amplifiers. The signals derive from neuron activities in the human brain. After passing through and spreading on the skull and scalp, the signals greatly decay relative to their strength on the surface of the cortex and contain significant noise from muscle activities and the environment. However, thanks to the progress in development of hardware and signal processing methods, the quality of EEG signals and usability of EEG devices have been greatly improved. Neuroscientists found many useful features in EEG signals that are linked to sensory processing, attention, arousal, motor imagination, and error detection. EEG signals can be continuously measured from a person; therefore, they are very useful anchors to examine humans’ perceptions without interrupting their behavior or asking a person to engage in introspection. Numerous prior studies have proposed brain-machine interfaces using EEG signals, decoding a human’s intention to control the machine. However, because of the noisy nature of EEG signals, such decoding techniques are far less accurate than a human’s voluntary control.

**Figure 1:**
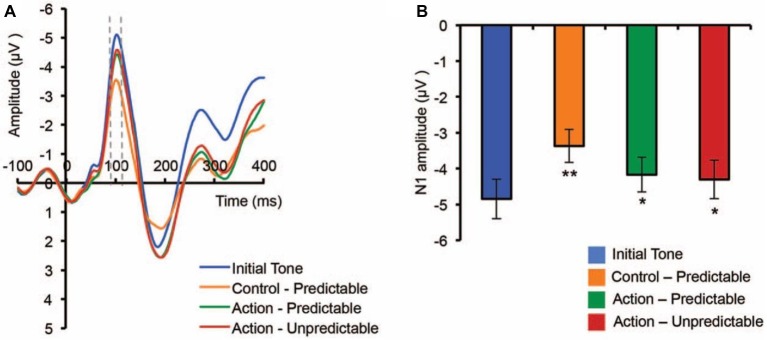
Grand-averaged ERPs across the Fz and FCz electrodes showing attenuated N1 response for an event that is caused by one’s action. **(A)** shows the EPRs time-locked to the onset of the one. **(B)** shows the amplitude of N1 in each condition. Original figure from Bednark et al. (2015). Permission was not required according to APA Permissions Policy.

Research on measuring individuals’ sense of agency using EEG is in its infancy. Neuroscientists are currently mainly employing hypothesis-driven approaches to determine candidate features in EEG signals that may be linked to the sense of agency. For example, previous studies reported that the N1 and P3 event-related components (ERPs), which are negative and positive potentials observed 100 and 300 ms after the onset of an action consequence, respectively, are attenuated in the condition when there is a sense of agency compared to the conditions when the sense of agency diminishes (Figure 1; Gentsch and Schütz-Bosbach, 2011; Kühn et al., 2011; Timm et al., 2014; Bednark et al., 2015). The explanation behind the attenuated ERPs is similar to that of sensory attenuation: processes for predicted sensory feedback may be centrally canceled, or the difference between the baseline (i.e., usually 200 ms before the onset of the event) and the peak of post-stimulus potentials is smaller due to pre-activation prompted by predicted events.

**Figure 2:**
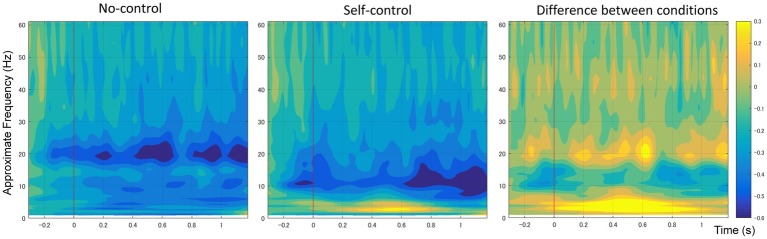
Average spectral power of 1–60 Hz frequency bands at the C3 electrode showing a suppression at the alpha-mu band in the self-control condition. Original figure from Wen et al. (2017a). The reproduction of the figure is permitted under the terms of the Creative Commons Attribution License (CC BY).

Few studies have examined how EEG signals may be linked to the sense of agency during continuous actions. During many daily circumstances such as driving, people receive a stream of feedback while continuously performing a series of actions. People may receive both positive and negative evidence of the sense of agency. When the accumulated evidence reaches a threshold, people conclude a sense of agency or non-agency. During such continuous action and feedback cycles, ERP for each single event may interfere and overlay with each other, making it impossible to measure. Instead, time-frequency analysis provides a better monitoring of mental status relating to the sense of agency. Wen et al. (2017a) and Kang et al. (2015) reported a suppression of alpha-mu rhythm linked to the sense of agency during movements (Figure 2; Kang et al., 2015; Wen et al., 2017a). Alpha-mu suppression is also called alpha-mu event-related desynchronization (ERD). ERD is a short-lasting and localized amplitude decrease of rhythmic activity and is frequently found in the alpha (8–12 Hz) and beta (12–30 Hz) rhythm during or prior to some behavior or mental state. It can be induced by motor preparation (Leocani et al., 1997; Cochin et al., 1999; Ramoser et al., 2000; Muthukumaraswamy et al., 2004; Pineda, 2005) and selective attention (van Winsun et al., 1984; Dujardin et al., 1993; Suffczynski et al., 2001; Polich, 2007). It has also been widely used in brain-machine interfaces to classify humans’ motor commands (e.g., move left vs. move right) (e.g., Doud et al., 2011). Regarding a sense of agency during continuous movements, the alpha-mu suppression may reflect the conversion from explorative action to exploitative action, as when people feel a sense of agency they can then better plan and prepare for subsequent actions (Wen et al., 2017a). In short, alpha-mu suppression during the experience of agency may reflect changes in the human internal motor system and therefore may be useful as a feature to decode the sense of agency.

Hypothesis-driven approaches provide clear theoretical accounts for phenomena but do not identify an inevitable phenomenon. In laboratory experiments, researchers designed conditions with and without the sense of agency, compared the EEG signals between the conditions, and reported the differences in some proposed features. However, this does not mean that if we observe a difference in these EEG features, we can make certain conclusions about the human sense of agency. The same feature may also be influenced by numerous other cognitive functions. It is very challenging to decode the sense of agency from brain activities, as the experience of agency is integrated from multiple cognitive processes, both at high and low levels. A recent study reported a high accuracy in decoding subliminal prediction errors (Ganesh et al., 2018). Prediction error is known as a dominant contributor to the sense of agency. Therefore, Granesh et al.’s (2018) work implied a potentially feasible direction to decode the experience of agency through probing it with a subliminal stimulus.

## How to Measure the Sense of Agency in Driving Automation

Given the circumstances of driving, the methods used in psychological experiments to measure the sense of agency face certain obstacles. Subjective judgment (e.g., binary judgment, agency rating) is the easiest way to measure agency. However, as discussed in section “Measurement of the Sense of Agency,” subjective judgment can be easily biased or influenced by the categorization of experimental conditions. Besides these issues, asking a driver to report his/her sense of agency might interrupt driving. Therefore, it may be useful for measuring the overall sense of agency after each driving trial, but less applicable in a situation where the sense of agency dynamically changes.

Sensory attenuation and the intentional binding effect are even more difficult to apply in a driving task, principally because these two methods are mainly designed for single instead of continuous feedback. Intentional binding also requires an interval between an action and its effect. Such delay is unnatural for a driving task and can greatly disturb driving performance.

To measure the sense of agency in driving automation, especially when the sense of agency may dynamically change, we suggest three potentially useful approaches. The first approach is to use physiological signals to decode the sense of agency. As introduced in section “Physiological Signals Linked to the Sense of Agency,” studies in neuroscience and psychology have reported some useful features of the signals linked to a sense of agency. However, physiological signals were usually used as dependent variables in most of the previous studies, and as these features can be also influenced by other extraneous variables that were controlled in the previous studies, they might not provide high accuracy as indices of agency if the extraneous variables are not sufficiently controlled. Nevertheless, future studies to decode agency using physiological signals would be necessary for this approach.

The second approach is to use attention as a proxy for the sense of agency. Previous studies showed that the sense of agency captures human attention (Salomon et al., 2011, 2013; Kumar et al., 2015). A recent study using steady-state evoked potentials (SSVEP: a flicker-triggered synchronized EEG potential; see Norcia et al., 2015 for a review) showed that people implicitly pay more attention to an object that they can better control (Wen et al., 2018). This study indicates that attention, which can be measured by eye gazing behavior, SSVEP, and many other behaviors, may be useful for monitoring the sense of agency.

Finally, control-motivated action may be another useful proxy for the sense of agency (Wen, 2019). Previous research in psychology showed that the sense of agency motivates actions (Eitam et al., 2013; Karsh and Eitam, 2015; Karsh et al., 2016). In order words, the stronger people feel a sense of agency, the more they may intend to exert control. In driving automation, the driver’s steering, gaze, and foot behavior can be potentially associated with the sense of agency. However, such behaviors can also be affected by environmental conditions, decision-making, and the driver’s reliance on driving assistance. A probe stimulus that creates a small prediction error and may trigger recovery behavior (to reduce prediction error) may be useful to probe the sense of agency. Such recovery behavior would be triggered only when people still feel a sense of agency and are monitoring potential prediction errors.

## Sense of Agency in Joint Control

In the past decade, joint actions have become a research interest in social interaction. Joint actions affect both the predictive and postdictive processes underlying the sense of agency. Because actors in joint actions do not have access to co-actors’ intentions, motor commands, or sensory feedback, the accurate matching between sensory feedback and the self’s and co-actors’ action becomes impossible, and the attribution of control is vague. Gallotti and Frith (2013) proposed the existence of a “we-mode” in social interaction, in which people automatically track co-actors’ attention (Samson et al., 2010; Böckler et al., 2012), performance (Sebanz et al., 2005), and beliefs (Apperly and Butterfill, 2009; McClure et al., 2010). van der Wel (2015) examined the influence of “we-mode” on the sense of agency when an actor and co-actor were either a decider or a follower. The results showed that when the actor and co-actor shared an intention, the sense of agency was high for both the decider and the follower. By contrast, conflicting intentions broke down the “we-mode” and shifted toward “I-mode” processing, resulted in a high sense of agency for the decider but a low sense of agency for the follower (van der Wel, 2015). Furthermore, van der Wel et al. (2012) examined the sense of agency experienced when people learned a novel motor skill in individuals and dyads, and found that the sense of agency did not change when people first learned the skill individually and then shifted to joint control, but the sense of agency greatly increased when whey performed individually after having performed with someone else, although there was no significant difference in performance between the individual and joint control conditions. This result indicated that when joint control is a baseline, individual control results in a higher sense of agency. This is likely an influence of social interaction in judgment of agency. By contrast, when individual control is treated as a baseline, the sense of agency in joint control is probably most affected by joint task performance.

During SAE level 1–2 driving automation, a driver and a system share control over the vehicle. For example, when the driver is steering, the moving direction of the car may be modified by the system for safety and performance purposes. Dewey et al. (2014) examined individuals’ judgment of control in such control-sharing conditions. Participants used joysticks to keep a cursor centered on a moving target with a co-actor. The sense of agency decreased in the condition of shared control compared to the condition of controlling the cursor alone. However, *predictable* effects produced by cooperative co-actors also increased the sense of agency, even when the effects (i.e., cursor movements) were not correlated with self-generated joystick movements. This indicates that the sense of agency during joint actions is evaluated with respect to both egocentric and group-level intentions (Dewey et al., 2014).

Furthermore, Wen et al. (2015c) designed a computer assistance program in a dot moving game, in which the computer only ignored participants’ erroneous commands. The results showed that when there is a delay in response in the game, making the game very difficult, the computer assistance greatly increased participants’ sense of agency compared with the condition when all participants’ commands were executed. Wen et al.’s results showed that the influence of goal achievement plays a dominant role in the sense of agency in the complex control condition (Wen et al., 2015c). Later, researchers from another group replicated Wen et al.’s (2015c) finding, adding an extended finding that this effect of assistance held even when the participants were explicitly aware of the assistance (Inoue et al., 2017). In summary, the above studies showed that shared control weakens the sense of agency but that the sense of agency can be maintained at a high level if the co-actor shares the agent’s intention and the joint action achieves a good performance.

## Sense of Agency in Robotics

When interacting with robots, feedback is usually more complicated than in the case of simple machines, because robots are designed to execute tasks automatically. Below, we discuss the following aspect of the sense of agency in robotics: the sense of agency over the robot, the sense of agency over external events *via* the robot, and the sense of agency over external events when collaborating with the robot.

The sense of agency over the robot probably relies on similar processes as the sense of agency over external events: whether the robot acts as predicted, and whether the task performance matches an individual’s prediction and expectation. Usually the sense of agency can be improved if a robot is responsible and performs as a user intended. A recent study on brain-computer interfaces (BCI) reported that when a user controlled a humanoid robot through BCI-SSVEPs (SSVEPs: steady-state evoked potentials), the additional feedback of audio-visual synchrony between a footstep sound and an actual humanoid’s walk reduced the time required for steering the robot and increased the feeling of control over the robot (Tidoni et al., 2014). This study implicated the importance of multisensory feedback during the remote control of a humanoid robot, especially when the control is complicated and difficult. In addition, a recent study reported an enhanced intentional binding effect when people tapped on their own arms compared to tapping a button, indicating that bodily feedback may enhance the sense of agency when interacting with machines (Coyle et al., 2012).

Decoding operators’ intention is crucial for providing operators with a sense of agency over external events *via* a robot. Recent studies showed that when a BCI successfully decoded operators’ motor commands and provided corresponding visual feedback of the movement of a humanlike robot hand (or virtual hand), people not only experienced a sense of agency over the robot hand, but also felt an illusion of bodily ownership over it (Perez-Marcos et al., 2009; Slater et al., 2009; Alimardani et al., 2013). The sense of agency in such cases was high, because the system provided both proximal and distal outcomes consistent with the user’s intention (Metcalfe et al., 2013; Vinding et al., 2013). During the embodiment of a robot, people usually do not feel that they are sharing control with the robot; instead, they feel that the robot hand is a part of their body and is under the full control of their own (in ideal decoding conditions).

In many human-machine interactions, people share control with machines, and, usually, are aware of that fact. Recalling the studies on the sense of agency in joint action, the sense of agency is usually weakened by sharing control with a co-actor. The presence of another human (Beyer et al., 2017) or robot (Ciardo et al., 2018) reduces the sense of agency over external events, even when people actually have full control. In short, in ordinary circumstances, the sense of agency is usually lower when people share control with robots, compared to the condition when people perform actions alone.

Shared control is often promoted for purposes of safety and efficiency. Machines remove a proportion of control from users, while ensuring more reliable and safer control of outcomes. The sense of agency and system performance seem like a trade-off. However, Wen et al. (2015c) showed that the sense of agency and performance can also be compatible. Specifically, in Wen et al.’s (2015c) study, the computer assistance was designed to ignore user’s erroneous commands in a goal-directed motor task. The results showed that computer assistance significantly improved the task performance, and, at the same time, significantly improved the sense of agency compared with the condition when the user’s commands were all executed. This result indicates that the dominance of each of the various processes underlying the sense of agency may change in different circumstances, and semi-automation that promotes the dominant process (e.g., task performance) but slightly impairs the non-dominant process (e.g., action-outcome comparisons) can achieve both good task performance and strong sense of agency at the same time. In a recent study, a semi-automatic system for teleoperation of a construction machine combining ideal working trajectory with the operator’s manual trajectory maintained the operator’s sense of agency at a high level, similar to the level of manual control, while improving the performance (Tanimoto et al., 2017). More importantly, Tanimoto et al. (2017) also found that the sense of agency was greatly weakened if the semi-automatic system performed a goal-directed assistance rather than a trajectory assistance. This is consistent with findings in cognitive science that both proximal and distal outcomes are important for the sense of agency (Metcalfe et al., 2013), as the goal assistance provided poor proximal feedback. In summary, an ideal assistance aiming for compatibility between performance and sense of agency should provide both proximal feedback to maintain users’ sense of agency and distal feedback that matches users’ intention.

## Sense of Agency in Automation

**Figure 3:**
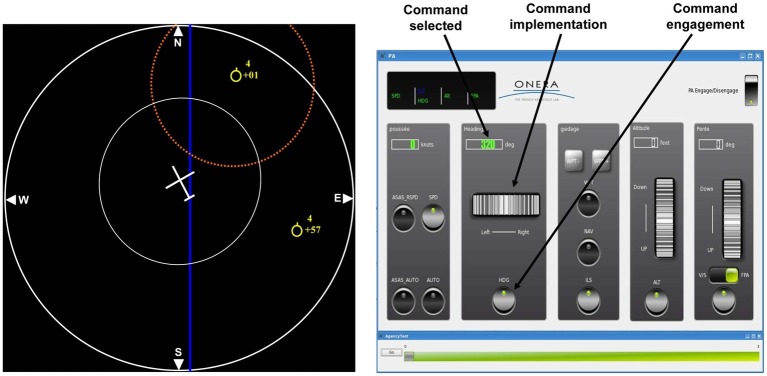
Experimental setup with the navigation display and the autopilot interface. Original figure from Berberian et al. (2012). The reproduction of the figure is permitted under the terms of the Creative Commons Attribution License (CC BY).

A French group used the intentional binding effect to examine how the degree of automation influenced the sense of agency in an aircraft supervision task (Berberian et al., 2012). Specifically, participants performed an aircraft navigation task in a simulator to change the aircraft’s horizontal trajectory to avoid conflict (Figure 3). Participants chose a heading command when the screen showed a conflict alarm, implemented the command using a scroll wheel, and finally executed it by pressing an engagement button. After the execution of the command, feedback concerning the success of the action was presented to the participants. Participants estimated the temporal interval between the keypress to engage the command and the feedback indicating success. The procedure of deciding, implementing, and executing the command to resolve a conflict was automated at four different levels. The results revealed a gradual increase of interval estimation with the increasing level of automation. In other words, the intentional binding, as an implicit of sense of agency, became weaker when the procedure was more automated (Berberian et al., 2012). Berberian et al.’s (2012) study was the first to show that automation not only affected explicit agency attribution, but also weakened the perception of the causal relationship between an action and its effect. That is, automation not only weakens the attribution of one’s own agency, but also influences people’s perception of the causes of events.

In the case of driving automation, the driver may be relieved from cognitive load and fatigue from driving tasks but may also stop monitoring the environment and preparing for action. A previous study that examined emergency braking during the use of cruise control found that the reaction time for braking was significantly longer when people used cruise control rather than controlling the speed of the vehicle manually (Jammes et al., 2017). This result was probably due to a lack of motor preparation and the cost of task shifting. The effect of cruise control was larger in older participants than in younger participants (Jammes et al., 2017). Furthermore, research in cognitive science showed that the sense of agency greatly influences attention allocation. People efficiently monitor events that are relevant to themselves (i.e., under their control), but do not pay much attention to events that are out of their control (Wen and Haggard, 2018). In the case of driving a car, if people have a sense of agency over the car, their monitoring of the car and dynamic changes in the environment would be more sensitive and efficient. If people do not have a sense of agency or become too reliant on the car’s self-control, delay or even dismissal of intervention responses by the driver is highly possible. In summary, in the case of human-machine joint control, if the human party’s monitoring and intervention are still required, the sense of agency is critical to making such joint control fluent and efficient.

## The Sense of Agency in Driving Assistance

While the research on driving automation in engineering and computer science has mainly focused on challenges in technique, some research has indeed shed light on human behavior and performance during driver-automation joint control. Yun et al. (2018) directly measured the driver’s sense of agency during assisted and automated driving in a driving simulator using an agency questionnaire. The results showed that even under the assisted driving condition, under which the simulator only intervened in steering when the vehicle went away from the road, the sense of agency was significantly lower than under the manual driving condition (Yun et al., 2018). The reduced sense of agency under the driving assisted condition was probably due to the fact that the drivers could easily detect the intervention of the system, and reflected the error detection in their agency report. Furthermore, research on the human-machine interface showed that driving support indeed decreased the driver’s control activity (Mulder et al., 2012) and is linked to driver disengagement (Navarro et al., 2016), indicating the loss of a sense of agency. This lack in a sense of agency can cause problems when the driver needs to resume manual control (Navarro et al., 2016).

According to psychological research on joint control, a “we-mode” may be essential to preserve the sense of agency during driving assistance (van der Wel et al., 2012; van der Wel, 2015). To form the “we-mode,” the system should not take over all the control from a driver. Instead, it is critical to detect the driver’s intention and assist in its fulfillment without disturbing the sense of agency. Recent research by engineers has sought to determine the driver’s intention through the use of vehicle sensors (Kuge et al., 2000; Berndt and Dietmayer, 2009; Wang et al., 2018b). For example, one research group tried to estimate the driver’s steering intention through gaze behavior, and provided haptic guidance to indicate the level of assistance during driver-automation shared control (Wang et al., 2018a,b). Their results showed that the correlation between gaze and steering movements decreased when the level of automation increased (Wang et al., 2018b). This may indicate a lack of control motivation when drivers did not have much control over the vehicle. The group further showed that the driver’s reliance on the assistance system greatly affected lane changing performance, especially in the case of a system failure (Wang et al., 2018a). This means that the extent to which people actively decide to take over control of the vehicle is important in driving performance, not only how they passively feel about the control. Although research in engineering does not always directly focus on the human sense of agency, the findings regarding human behavior, attention, and performance provide many insights into the importance of the sense of agency and how it might change during driving assistance. Identifying the intention of the human driver and providing less obtrusive assistance represent a necessary step in future research on driver-automation collaborative driving systems.

In this paper, we discussed the importance of the sense of agency in driving automation. Two main questions remain to be solved in future research on this topic: (1) how to monitor the sense of agency when people use driving automation and (2) how to design the driving automation to ensure the compatibility of driving safety and the sense of agency. These questions are important for human-machine interaction in driving automation when the human is considered as a *driver* or *supervisor* instead of a passenger. Here, we suggest two directions for further research. The first is to monitor the sense of agency in real-time when using driving automation. Further study on measuring the sense of agency using onboard vehicle sensors, such as cameras and force sensors at steering wheel, brake, and accelerator, is necessary. The second is to design the driving automation system by carefully measuring the sense of agency in the laboratory using all possible sensors and methods (see section “Measurement of the Sense of Agency”) in different driving circumstances. Having said this, the establishment of the model of human sense of agency in driving automation is important. With such a model, researchers can estimate the threshold of sense of agency in different situations, and provide a good solution that ensures both safety and human agency according to the situation.

## Conclusion

In this review, we focused on the role of the sense of agency in driving automation within its current commercial context, in which driver-automation joint control is required. Usually, the more reliable the driving automation, the less the driver might be engaged. The lack of a sense of agency is considered a reason for driver disengagement, which can be problematic when the driver’s decision-making is required. For research on driving automation, we suggest first that monitoring drivers’ sense of agency under different circumstances of use of driving automation is important. Physiological signals, attention (e.g., gaze behavior), and control-motivated actions (e.g., prediction-error probed actions; see details in section “How to Measure the Sense of Agency in Driving Automation”) may be more useful to measure the sense of agency than the traditional methods used in psychological experiments such as self-report, sensory attenuation, and intentional binding effect. Second, we suggest that in order to maintain a sense of agency at a good level, generating a “we-mode” is essential for driver-automation joint control. To achieve this, techniques decoding the driver’s intention are critical. Recent research in engineering is attempting to do so using onboard vehicle sensors (Kuge et al., 2000; Berndt and Dietmayer, 2009; Wang et al., 2018b). However, in most of the previous studies, vehicles usually executed steering/accelerating/decelerating for the driver after the recognition of the driver’s intention, which might result in the driver’s loss of sense of agency over the vehicle. It remains unsolved how to combine the driver’s and the automation system’s control over the vehicle to optimize both driving performance and the sense of agency. Indeed, combining the knowledge and methodologies of cognitive science and techniques such as sensing and modeling in engineering may yield a good solution for a driver-automation collaborative driving system that ensures efficient joint control characterized by high safety, performance, and cognitive efficiency.

## Author Contributions

WW drafted the manuscript. WW, YK, and HA reviewed and revised the manuscript.

### Conflict of Interest

The authors declare that the research was conducted in the absence of any commercial or financial relationships that could be construed as a potential conflict of interest.

## Footnotes

^1^SAE International, 2014: “*Dynamic driving task* includes the operational (steering, braking, accelerating, monitoring the vehicle and roadway) and tactical (responding to events, determining when to change lanes, turn, use signals, etc.) aspects of the driving task, but not the strategic (determining destinations and waypoints) aspect of the driving task.”

^2^SAE International, 2014: “*Driving mode* is a type of driving scenario with characteristic dynamic driving task requirements (e.g., expressway merging, high speed cruising, low speed traffic jam, closed-campus operations, etc.).” *Driving mode-specific execution* refers to the execution of steering and acceleration/deceleration in this driving mode.
